# Anatomical and CBCT-Based Evaluation of the Mental Foramen in Korean Adults: Clinical Implications for Implant Surgery and Mental Nerve Block

**DOI:** 10.3390/diagnostics15243109

**Published:** 2025-12-07

**Authors:** Yong-Ho Kim, Mi-Sun Hur

**Affiliations:** Department of Anatomy, Daegu Catholic University School of Medicine, Daegu 42472, Republic of Korea

**Keywords:** mental foramen, mandible, gingival margin, cone-beam computed tomography (CBCT)

## Abstract

**Background:** Precise localization of the mental foramen (MF) is essential to avoid mental nerve injury during implant placement, osteotomy, periapical surgery, and regional anesthesia. However, MF morphology and canal orientation show population-specific variability, and comprehensive morphometric data combining cadaveric dissection and CBCT analysis remain limited in Koreans. This study aimed to provide clinically applicable MF reference values for Korean adults. **Methods:** Thirty-two hemimandibles from 16 dentate Korean cadavers were examined through direct anatomical dissection. MF position relative to teeth, shape, vertical and horizontal diameters, and distances to the gingival margin, inferior border, and mandibular midline were measured. CBCT imaging of 12 hemimandibles was used to assess the internal trajectory and opening direction of the mental canal. **Results:** The MF was most frequently located below the second premolar (75%), followed by the P2–M1 region (15.6%). Round foramina (62.5%) were more common than oval forms. Mean distances from the MF to the gingival margin, inferior border, and midline were 16.6 mm, 15.5 mm, and 26.5 mm, respectively. The mean horizontal diameter of the MF was 3.0 mm, and the mean vertical diameter was 2.2 mm. CBCT analysis revealed two emergence patterns—posterolateral (50%) and lateral (50%). **Conclusions:** This study identified the positional characteristics, diameters, and canal emergence patterns of the MF in a cadaveric sample using dissection and CBCT. The findings regarding MF location, dimensions, and the opening direction of the mental canal provide practical anatomical information that may support safer implant placement, mental nerve blocks, and anterior mandibular surgery.

## 1. Introduction

The mental foramen (MF) is a critical anatomical landmark, and its precise localization is essential prior to dental implant placement and osteotomy to prevent associated complications [1,2]. Accurate identification of the MF is also indispensable when administering regional anesthesia of the incisive nerve—the other terminal branch of the inferior alveolar nerve—and when performing periapical surgery in the premolar and molar regions to avoid inadvertent nerve damage [3]. The mental nerve, the terminal branch emerging through the MF, supplies sensation to the lower lip, buccal/oral mucosa, and the skin of the chin ventral to the mental foramen [4,5,6]. Injury to the MF or mental nerve—or inadequate anesthesia in this region—can lead to various sensory disturbances, including paresthesia, hypoesthesia, hyperesthesia, dysesthesia, or even complete anesthesia of the lower lip, gingiva, and adjacent skin and mucosa. Such neurosensory deficits may arise from direct violation of the mandibular canal or foramen during osteotomy, implant impingement, or pressure caused by edema, hematomas, scars, or dental injections. Therefore, accurate identification of the MF, assessment of the potential anterior loop, and establishment of an adequate safety zone (typically at least 2 mm between the implant and the coronal aspect of the nerve) are essential to prevent iatrogenic nerve injury [7].

Despite its importance in clinical practice, the MF demonstrates considerable interindividual variability in its position, shape, dimensions, and opening direction, including the course of the mental canal [8]. Previous studies across various populations and methodological approaches have consistently demonstrated that the MF is most commonly located in the P2 or P1–P2 region [9,10,11,12,13,14,15,16,17,18,19,20]. Hur et al. (2008) [21] reported the anatomical relationship between the depressor anguli oris and the MF, for the most effective site for injecting botulinum toxin type A when treating the labiomandibular fold, whereas Hwang et al. (2005) [22] advised that the level of sliding mentoplasty should be placed at least 4.5 mm inferior to the MF to avoid injury to the inferior alveolar nerve. Furthermore, previous anatomical studies have demonstrated substantial variability not only in the configuration of the anterior loop of the mandibular canal [23] but also in the branching patterns and intraosseous course of the mental nerve, highlighting the need for careful assessment of the MF region during clinical procedures [24]. Moreover, CT-based analyses have demonstrated that minor spatial variations near the mandibular canal can affect neurosensory recovery, supporting the need for meticulous anatomical assessment in the MF area as well [25]. This anatomical variability increases the risk of unexpected neurovascular injury and underscores the need for accurate, population-specific morphometric data. However, there remains a paucity of data in the Korean adult population, particularly combining gross dissection and CBCT analysis.

Therefore, this study aimed to provide clinically applicable, population-specific morphometric reference data for the MF in Korean adults by integrating direct cadaveric dissection and CBCT analysis.

## 2. Materials and Methods

### 2.1. Specimens and Anatomical Measurements

A total of 32 hemimandibles from 16 cadavers (8 males and 8 females; 77.4 years old; range 56–92 years) were analyzed. Specimens with missing teeth around the MF were excluded. The skin and muscles surrounding the MF were carefully removed, and the mental neurovascular bundle emerging from the MF was excised to clearly expose the shape and location of the MF. The items observed and measured were as follows, and all measurements were taken with reference to the external surface of the mandible at the level of the MF.

Measurements were taken using a digital caliper. The following parameters were evaluated:

1. The positional relationship between the MF and the teeth.
2. The shape and the horizontal and vertical diameters of the MF.
3. The distance from the superior margin of the MF to the gingival margin.
4. The distance from the superior margin of the MF to the inferior border of the mandible.
5. The distance from the medial margin of the MF to the mandibular midline.

### 2.2. Mandibular CBCT Imaging and Mental Foramen Analysis

Among the 16 cadavers, 12 hemimandibles from 6 cadavers were used for CBCT analysis. Cone-beam computed tomography (CBCT) scans of the mandible were obtained using a Green X 3D imaging system (Vatech, Hwaseong, Republic of Korea) to evaluate the morphology and opening direction of the mental foramen. The DICOM datasets were imported into Mimics software (version 21.0; Materialise, Leuven, Belgium) to generate serial coronal, axial, and sagittal sections for three-dimensional assessment of mandibular morphology and mental canal orientation. The course and direction of the mental canal leading to the MF were also identified.

### 2.3. Ethical Approval and Informed Consent

All cadavers were legally donated to the Daegu Catholic University School of Medicine for education and research under legal body donation agreements. All cadavers used in this study were legally donated to the university through informed consent by the donors and/or their next of kin. The study was conducted in accordance with the principles of the Declaration of Helsinki. The study protocol was reviewed and approved by the Institutional Review Board of Daegu Catholic University (CR-24-103; Approval date: 29 August 2024). Informed consent for body donation for education and research was obtained from the donors and/or their next of kin.

## 3. Results

### 3.1. Morphology and Location of the MF

**Figure 1:**
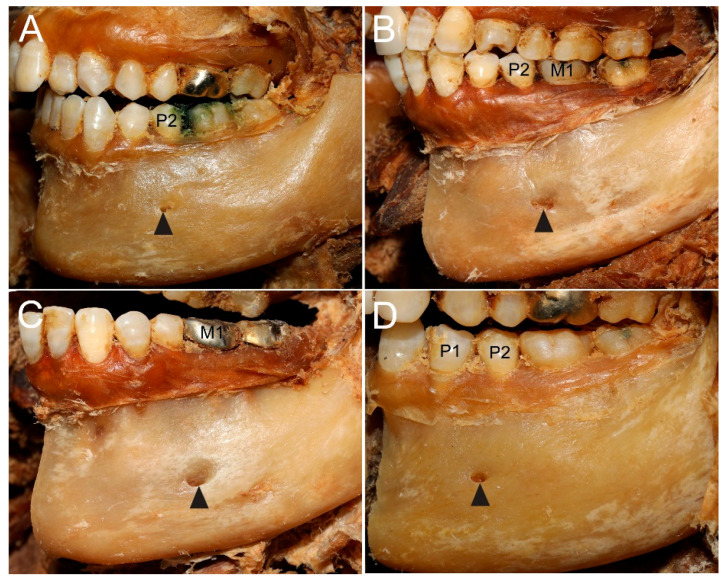
Anatomical variations in the location of the mental foramen (MF) relative to the mandibular teeth. (**A**) MF (arrowhead) located directly below the second premolar (P2), the most common pattern. (**B**) MF (arrowhead) located between the second premolar and first molar (P2–M1). (**C**) MF (arrowhead) located below the mesial root of the first molar (M1). (**D**) MF (arrowhead) located between the first and second premolars (P1–P2).

The MF was most commonly located in the region of the second premolar (P2)—below it in 24 of 32 specimens (75.0%), between the second premolar and first molar (P2–M1) in 5 specimens (15.6%), below the mesial root of the first molar (M1) in two specimens (6.3%), and between the first and second premolars (P1–P2) in one specimen (3.1%) (Figure 1). In males (16 specimens), the MF was located below P2 in 14 specimens and in the P2–M1 region in 2 specimens. In females (16 specimens), it was located below P2 in 10 specimens, in the P2–M1 region in 3 specimens, below the mesial root of the first M1 in two specimens, and in the P1–P2 region in one specimen. No accessory mental foramina were observed.

The mental foramina (MFs) were symmetrical in 9 of 16 cadavers (56.3%), with both sides located below the second premolar (P2). Asymmetry was observed in 7 cadavers (43.8%). Among the asymmetric cases, the most common pattern was a P2 position on one side and a P2–M1 position on the other (4 cadavers, 25%). Less frequent patterns included a P1–P2 position on one side and a P2 position on the other (1 cadaver, 6.3%), an M1 position on one side and a P2 position on the other (1 cadaver, 6.3%), and a P2–M1 position on one side and an M1 position on the other (1 cadaver, 6.3%).

**Figure 2:**
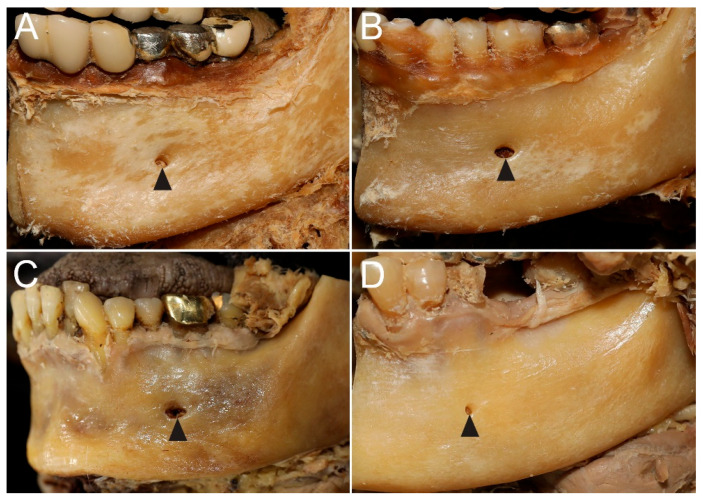
Morphological variations in the shape and diameter of the MF. (**A**) A round MF (arrowhead) with a moderate horizontal diameter. (**B**) An oval MF (arrowhead) with a relatively larger horizontal diameter. (**C**) A specimen with the largest MF (arrowhead), measuring 4–5 mm. (**D**) A specimen with a small MF (arrowhead), measuring 1–2 mm.

The MF was predominantly round (20 specimens, 62.5%) or oval (12 specimens, 37.5%). The horizontal diameter (3.0 ± 0.8 mm; range, 1.2–4.9 mm; males: 2.7 ± 0.6 mm; females: 3.2 ± 1.0 mm) was greater than the vertical diameter (2.2 ± 0.5 mm; range, 1.1–3.1 mm; males: 2.1 ± 0.5 mm; females: 2.3 ± 0.6 mm). The MF exhibited the largest horizontal diameter—measuring 4–5 mm on both sides—in two female cadavers. In these cadavers, the MFs were located below the M1 in two specimens, in the P2–M1 region in one specimen, and below the P2 in one specimen. In contrast, a smaller horizontal diameter of 1–2 mm was found in three specimens from one female and one male cadaver. In these cases, the MFs were located below P2 in two specimens and in the P1–P2 region in one specimen (Figure 2). The internal opening of the mental canal within the mandible was smaller than the external opening, indicating a tendency for the MF to widen as it exited toward the outer surface.

The mean distances were 16.6 ± 2.2 mm (range: 11.7–22.3 mm; males: 16.9 ± 1.4 mm; females: 16.2 ± 2.8 mm) from the superior margin of the MF to the gingival margin; 15.5 ± 1.6 mm (range: 12.7–18.4 mm; males: 16.5 ± 1.4 mm; females: 14.5 ± 1.2 mm) to the inferior border of the mandible; and 26.5 ± 2.3 mm (range: 20.8–31.2 mm; males: 26.8 ± 1.9 mm; females: 26.2 ± 2.6 mm) to the midline. The mean right–left asymmetry was 1.0 mm (range: 0.0–2.4 mm) for the gingival margin, 0.8 mm (0.0–2.2 mm) for the inferior border of the mandible, and 2.4 mm (0.0–10.5 mm) for the midline.

### 3.2. CBCT-Based Analysis of Mental Canal Opening Direction

**Figure 3:**
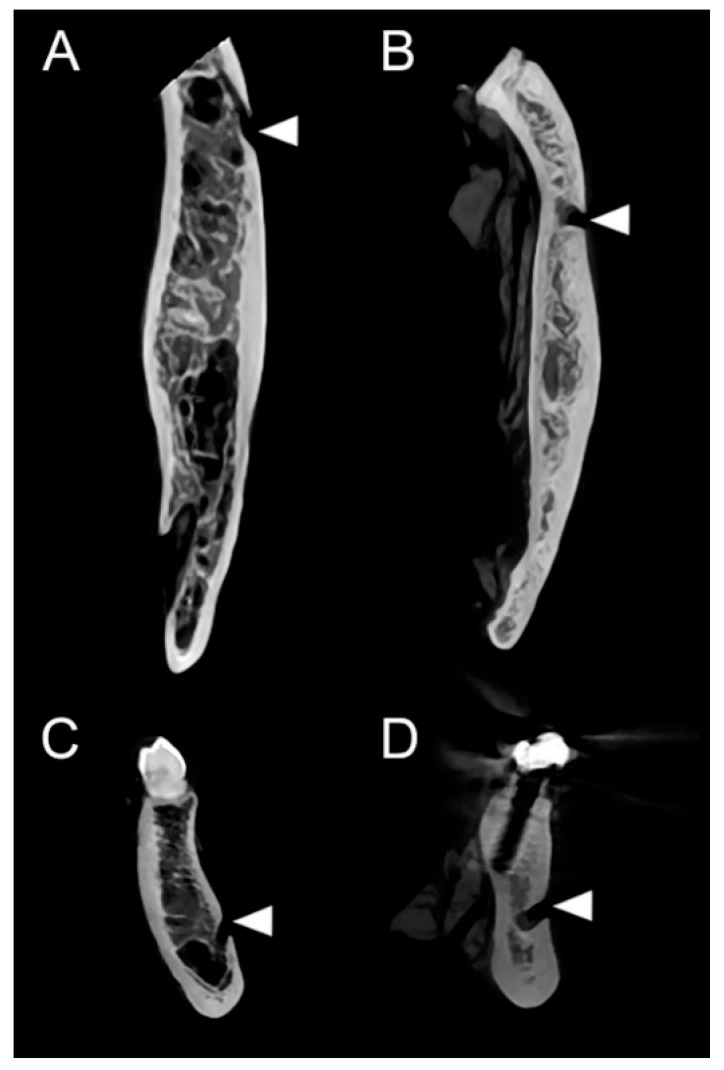
CBCT-based analysis of the opening direction of the mental canal. (**A**,**C**) Axial (**A**) and coronal (**C**) images demonstrating the posterolateral opening type, with the mental canal coursing posterolaterally toward the mental foramen (MF). (**B**,**D**) Axial (**B**) and coronal (**D**) images demonstrating the lateral opening type, in which the mental canal courses almost laterally before exiting through the MF. Arrowheads indicate the opening of the mental canal.

The opening direction of the mental canal was classified into two distinct patterns: a posterolateral type and a lateral type. Among the 12 specimens, six demonstrated a posterolateral opening. The degree of posterolateral inclination varied considerably, ranging from a subtle posterolateral deviation to a markedly oblique posterior–lateral trajectory. In contrast, the remaining six specimens exhibited a lateral opening pattern, in which the mental canal maintained an almost straight course before reaching the MF. Overall, both patterns were equally represented, with the posterolateral type showing some variability in its angulation (Figure 3; Videos S1–S4).

## 4. Discussion

The present investigation was designed to allow direct anatomical verification of MF morphology and to obtain precise morphometric data based on the external mandibular surface. Unlike CBCT-based analyses that commonly adopt the alveolar crest as the superior reference point, this study used the gingival margin, which—although affected by age-related alveolar bone resorption—offers a more clinically accessible and relevant landmark for surgical and anesthetic procedures in living individuals. This methodological approach enhances the practical applicability of the findings. In addition, CBCT imaging was incorporated to visualize the course and emergence patterns of the mental canal, enabling detailed assessment of both its trajectory and exit direction. Together, these analyses provide comprehensive reference data applicable to clinical procedures involving local anesthesia, implant placement, and maxillofacial surgery in the mental region.

Although the present cadaveric analysis in Koreans similarly identified P2 as the predominant location, several morphometric and positional characteristics differed notably from previous radiologic and cadaveric findings (Table 1).

Regarding MF location, several previous studies consistently reported the P2 position as the most common, with the P1–P2 region frequently cited as the second most prevalent site [11,13,14,15,17,18]. In the present study, while the P2 position remained the most frequent (75%), the second-most common location was the P2–M1 region (15.6%), which diverges from the patterns described in the above studies. Sex-specific analysis additionally revealed broader positional variability in females, who demonstrated four MF locations, whereas males exhibited predominantly two patterns (P2 and P2–M1). No accessory MFs were identified.

Differences were also evident in morphology and size. Previous CBCT studies have consistently reported the oval type as the dominant MF shape—74.3% in the Iranian population [26] and 73–76% in the Saudi population [19]—with round foramina accounting for only 24–27% of cases. In contrast, Goyushov et al. (2018) [16] reported a reversed pattern, with the round type being more prevalent (61.1% on the left and 62.1% on the right) and the oval type comprising only 26.7–27.9%. Similarly, a recent study from the Yemeni population by Algabri et al. (2025) [20] further supported this pattern, reporting round foramina as the most common shape (46.3%), followed by irregular types (38.2%). The present cadaveric findings aligned more closely with the latter, showing a predominance of the round type (62.5%), which occurred nearly twice as often as the oval type (37.5%). Such discrepancies among studies may reflect true population-specific variation.

The comparative data in Table 2 demonstrate substantial methodological and population-specific differences in mental foramen measurements reported across previous studies [10,12,13,17,18,19,26,27,28,29,30]. Previous studies used diverse superior reference landmarks—including the alveolar crest, CEJ, premolar cusp tip, and the bottom of the lower second premolar socket—which produced wide variability in MF–superior distances, ranging from 2.5 mm to 25.7 mm. Such inconsistency indicates the difficulty of establishing universally applicable vertical reference values and reflects fundamental differences in landmark selection rather than true anatomic variability.

In contrast, the present study adopted the gingival margin as the superior reference point, yielding an MF–superior distance of 16.2 mm. Although the gingival margin was used in this study as a visually accessible intraoral landmark, it is not a stable reference point because it may shift with aging, periodontal status, or tooth loss. For this reason, the MF–gingival margin distance should be interpreted as a supplementary observation rather than a primary anatomical reference. The mandibular inferior border remains the more reliable and fixed landmark for determining vertical MF position. Nonetheless, the gingival margin can offer practical utility in clinical settings, where it provides an immediately visible intraoral cue for approximating MF height during mental nerve block, implant drilling, or anterior mandibular surgery.

Regarding the inferior distance, earlier studies commonly reported MF–inferior border distances of 10.3–21 mm [10,12,13,17,18,19,27,28], whereas the present Korean cadaveric sample demonstrated a greater mean distance of 15.5 mm. This finding is consistent with previous Korean studies [13,27], which also tended to report comparatively larger values than those observed in other populations. This upward positioning suggests that Koreans may have a relatively more superiorly located MF within the mandibular body compared with other populations.

Notably, the MF–midline distance measured in this study (26.5 mm) closely approximated previous values reported in Chinese, Caucasian, and Iranian samples [10,12,28], indicating that the mediolateral position of the MF remains relatively conserved across populations despite differences in vertical metrics.

Additionally, the horizontal diameter in this Korean sample (3.0 mm) was smaller than those reported in Iranian, Egyptian, Turkish, and Caucasian groups (3.5–4.4 mm) [12,17,26,28,29]. A reduced horizontal diameter implies a narrower neurovascular canal, potentially limiting the buffering space between the mental nerve and the bony walls, thereby increasing susceptibility to thermal or mechanical injury during implant drilling, periapical surgery, or mental nerve anesthesia. This narrower canal dimension may also reduce the margin of safety during osteotomy or implant insertion, meaning that even small deviations in drill angulation or depth could more readily compromise the mental nerve in Korean patients.

Collectively, these findings demonstrate that MF morphology and vertical position show meaningful population-dependent variation, while mediolateral distance remains stable. The present study provides Korean-specific morphometric reference data that can refine safety guidelines for anterior mandibular procedures and support more precise, population-specific surgical planning.

The anterior loop is a continuation of the inferior alveolar nerve that extends forward beyond the mental foramen before looping back to exit through it [31]. Previous studies have demonstrated substantial variability in the presence and extent of the anterior loop of the mental nerve, with reported frequencies ranging from 11.8% to nearly 82% across populations [17,26,30,31,32,33,34,35,36,37]. CBCT studies have also described diverse emergence patterns of the mandibular canal—straight, anterior, and posterior trajectories—and several anatomical classifications such as the Y-, T-, and anterior-loop types further underscore the morphological heterogeneity of this region [15,32]. No distinct anterior loop was identified in the present Korean cadaveric sample. The subtle posterolateral deviation observed in some cases likely represents minor anatomical variation rather than a true looping morphology.

This study has several limitations. First, the sample size was relatively small (32 hemimandibles from 16 cadavers), which restricts the generalizability of the findings. Second, because the specimens were obtained from donated cadavers, the age distribution was inherently limited and consisted mainly of older adults, which may have influenced certain MF measurements. Third, although the distance from the MF to the gingival margin was included, the gingival margin is not a stable anatomical landmark and varies with age and periodontal condition; therefore, this parameter should be regarded as a supplementary observation rather than a primary reference. Fourth, not all specimens could be scanned using CBCT due to limited availability when the cadavers were acquired, preventing one-to-one comparison between dissection and CBCT findings for every hemimandible.

In conclusion, this study presents detailed morphometric data on the MF in Korean adult specimens, integrating gross anatomical dissection and CBCT imaging. Such data can inform safer surgical planning in dental implantology, mandibular osteotomies, and reconstructive procedures, and serve as a population-specific anatomical framework for establishing more accurate safety guidelines around the mental foramen in Korean patients.

## Figures and Tables

**Table 1:** Previous studies reporting the most common location of the mental foramen across different populations and methodological approaches.

Year	Authors	Population	Method	Most Common MF Location (%)
1976	Fishel et al. [9]	Caucasian	Radiograph	P1–P2 (70.4%)
1986	Wang et al. [10]	Chinese	Cadavers	P2 (59.0%)
2003	Ngeow and Yuzawati [11]	Malay	PR	P2 (69.2%)
2004	Neiva et al. [12]	Caucasian	Cadavers	P1–P2 (58%)
2006	Kim et al. [13]	Korean	PR	P2 (64.3%)
2008	Amorim et al. [14]	Brazilian	Cadavers	P2 (Rt: 71.4%, Lt: 68.1%)
2015	Khojastepour et al. [15]	Iranian	CBCT	P2 (Rt: 48.7%, Lt: 51.9%)
2018	Goyushov et al. [16]	Caucasian	CBCT	P1–P2 (49.2%)
2020	Shalash et al. [17]	Egyptian	CBCT	P2 (Rt: 67.9%, Lt: 43.8%)
2021	Ahmed et al. [18]	Sudanese	CBCT	P2 (40.5%)
2022	AlQahtani [19]	Saudi	CBCT + PR	P1–P2 (Rt: CBCT-48%, PR-52%) (Lt: CBCT-50.7%, PR-48%)
2025	Algabri et al. [20]	Yemeni	PR	P1–P2 (63.2%)
2025	Present study	Korean	Cadavers	P2 (75%)

Abbreviations: CBCT, cone-beam computed tomography; PR, panoramic radiograph; P1–P2, between the first and second premolars; P2, second premolar; Rt, right; Lt, left.

**Table 2:** Comparative summary of reference landmarks and morphometric measurements of the MF reported in previous studies and in the present cadaveric study. (Units: mm).

Year	Author	Population	Method	MF Diameter	Superior Reference Landmark	MF–Superior Reference Distance	MF–Inferior Border of the Mandible	MF (Anterior Border)–Midline
1986	Wang et al. [10]	Chinese	Cadavers		Bottom of the lower second premolar socket—MF superior border	2.5	14.7 (from the MF inferior border)	28.1
2004	Neiva et al. [12]	Caucasian	Cadavers	Horizontal 3.6 Vertical 3.5	CEJ—MF superior border	15.5	12.0 (from the MF inferior border)	27.6
2006	Kim et al. [13]	Korean	PR	—	Premolar cusp tip—MF superior border	During operation: 23.4 PR: 25.7	During operation: 14.3 PR: 16.5 (from the MF superior border)	—
2016	Lim et al. [27]	Korean	3D facial CT	—	—	—	21.0 (from the MF inferior border)	—
2016	Sheikh & Kheir [28]	Iranian	CBCT	Rt 3.6 Lt 3.6	—	—	Rt: 13.3 Lt: 13.4 (from the MF superior border)	Rt: 25.9 Lt: 25.5
2019	Çağlayan et al. [29]	Turkish	CBCT + US	Horizontal - CBCT: 4.4 - USG: 4.3 Vertical - CBCT: 3.3 - USG: 3.3	Alveolar crest—MF superior border	CBCT:14.2 US: 12.8	—	—
2020	Shalash et al. [17]	Egyptian	CBCT	Horizontal 3.5 Vertical 3.4	—	—	10.3 (from the MF inferior border)	—
2021	Ahmed et al. [18]	Sudanese	CBCT	Horizontal - Male: 3.6 - Female: 1.2 Vertical - Male: 3.1 - Female: 1.6	Alveolar crest—MF superior border	Male: 14.2 Female: 13.5	Male: 13.6 Female: 12.1	
2022	Nikkerdar et al. [26]	Iranian	CBCT	Rt 4.3 Lt 4.2	—	—	—	—
2022	AlQahtani [19]	Saudi	CBCT + PR	—	—	—	CBCT: 11.5 PR: 11.7 (from the MF inferior border)	—
2025	Cimen et al. [30]	Turkish	CBCT	Rt 2.1 Lt 2.0				
2025	Present study	Korean	Cadaver	Horizontal 3.0 Vertical 2.2	Gingival margin—MF superior border	16.2	15.5	26.5

Abbreviations: MF, mental foramen; CEJ, cemento–enamel junction; CBCT, cone-beam computed tomography; US, ultrasonography; PR, panoramic radiograph; Rt, right; Lt, left.

## Data Availability

The original contributions presented in this study are included in the article/Supplementary Material. Further inquiries can be directed to the corresponding author.

## Supplementary Materials

The following supporting information can be downloaded at: https://www.mdpi.com/article/10.3390/diagnostics15243109/s1, Video S1 (Video A): Axial, posterolateral opening type; Video S2 (Video B): Coronal, posterolateral opening type; Video S3 (Video C): Axial, lateral opening type; Video S4 (Video D): Coronal, lateral opening type.
